# Associations of Overweight, Obesity and Related Factors with Sleep-Related Breathing Disorders and Snoring in Adolescents: A Cross-Sectional Survey

**DOI:** 10.3390/ijerph14020194

**Published:** 2017-02-15

**Authors:** Yue Ma, Liping Peng, Changgui Kou, Shucheng Hua, Haibo Yuan

**Affiliations:** 1 Pulmonary Division & Sleep Center, The First Hospital of Jilin University, Changchun 130021, China; mayue205@163.com (Y.M.); plp640317@163.com (L.P.); shuchenghua@eyou.com (S.H.); 2 Department of Epidemiology and Biostatistics, School of Public Health, Jilin University, Changchun 130021, China; koucg@jlu.edu.cn

**Keywords:** SRBD, snoring, overweight, obesity, association

## Abstract

*Background*: Sleep-related breathing disorders (SRBD) have been identified as a major public health problem closely related to adolescent obesity. We aimed to estimate the prevalences of SRBD and snoring in adolescents in Changchun City, Northeastern China, and to evaluate the associated factors in this population. *Methods:* In total, 1955 adolescents aged 11–18 years were recruited in Changchun City using stratified cluster sampling. Parents and caretakers of children completed the questionnaires, which included demographic characteristics, anthropometric parameters and a pediatric sleep questionnaire (SRBD scale). Logistic regression was used to analyze the relationship between SRBD, snoring and other factors. *Results:* The prevalences of SRBD and snoring in our population were 3.7% and 3.3%, respectively, and the prevalences of overweight and obesity were 12.6% and 4.9%. Multivariate logistic regression showed that urban residence (OR = 2.356, 95%CI: 1.251–4.435) and post-term birth (OR = 3.275, 95%CI: 1.396–7.683) were significantly associated with SRBD. Preterm birth (OR = 2.255, 95%CI: 1.021–4.980) and parental education level of university and above (OR = 0.265, 95%CI: 0.083–0.850) were significantly associated with snoring. Overweight (OR = 2.063, 95%CI: 1.062–4.006) was also related to snoring. *Conclusions*: The prevalences of SRBD and snoring were similar to those reported in previous studies. Urban residence and post-term birth were important influencing factors for SRBD; overweight, highest parental education level (university and above) and preterm birth were key factors affecting snoring in adolescents.

## 1. Introduction

Obesity is a major public health problem that has attracted considerable public attention worldwide [1,2,3]. The prevalence of obesity in China was 12% in 2010 [4]. The global prevalence of obesity in children has been increasing, from 4.2% in 1990 to 6.7% in 2010 [5]. In China, the prevalence of overweight among children and adolescent reached 17.1% in 2011 [6].

Adolescent obesity is associated with significant comorbidities, including sleep-related breathing disorders (SRBD), such as habitual snoring, obstructive sleep apnea (OSA), upper airway resistance syndrome and hypoventilation [7,8,9]. Obesity-related anatomic risk factors include the enlargement of parapharyngeal fat pads, lateral pharyngeal walls, the tongue (including tongue fat), and total upper airway soft tissue. All of these factors can play an important role in the pathogenesis of OSA in adolescents [10]. In addition to obesity, lymphoid tissue may affect the development of SRBD in adolescents [11]. Furthermore, upper airway neuromuscular reflexes may be impaired in this population. 

Adolescence is a transitional period during which a variety of sleep problems can occur, and these problems should receive a greater amount of attention. Adolescence is also one of the fastest growing periods with high risk of SRBD [12]. Obese adolescents with SRBD may be at high risk for neurobehavioral deficits, which can affect their daytime functioning as well as their future skills and abilities [13]. Because of the distinct dietary patterns in different regions of China and the regional characteristics, the severity of obesity is greater in northeast China than in other Chinese regions. However, the association between obesity and SRBD among adolescents in northeast China has not been explored. The aim of the current study was to investigate the prevalences of SRBD and snoring among adolescents in Changchun City and to analyze the influencing factors affecting SRBD and snoring. 

## 2. Materials and Methods

### 2.1. Subjects

A cross-sectional survey was performed in Changchun City, the capital of Jilin Province in Northeast China. We randomly selected six middle schools (three in urban and three in rural areas) using stratified cluster sampling. A total of 1955 students aged 11–18 years were recruited to participate in the survey; subjects with a history of neuromuscular disease, craniofacial syndromes, cerebral palsy, sickle cell disease, mucopolysaccharide storage disease, or immunodeficiency were excluded. Participants were also excluded if they had mental or physical impairment severe enough to cause abnormal behaviors, including congenital disease, intellectual disability, and a psychiatric disorder, and if their parents could not understand the questionnaire [14]. The study was approved by the ethics committee of the First Hospital of Jilin University (Reference Number: 2013-031). Informed consent was obtained from the students and parents.

### 2.2. Data Collection

The investigation was conducted by the First Hospital of Jilin University in April 2016. Details about the survey were provided by interviewers who had received prior training, and parents and caretakers of subjects completed the questionnaire. Demographic characteristics (age, gender, educational level, ethnicity, residence, exercise, highest parental degree, birth history and BMI classification), anthropometric parameters (weight, height, waist circumference, neck circumference, abdominal circumference, and hip circumference) and a pediatric sleep questionnaire- the Sleep-Related Breathing Disorder (PSQ-SRBD) scale—were included in the questionnaire.

### 2.3. Pediatric Sleep Questionnaire—Sleep-Related Breathing Disorder Scale

PSQ-SRBD scale is used to assess sleep-related breathing disorders in children aged 2–18 years old and is administered by parents and caretakers. The PSQ-SRBD is composed of 22 items which are divided into three domains as follows: snoring (nine items), sleepiness (seven items) and behavior (six items). The response options for each item include “no”, “yes” and “do not know”, and the scores range from 0 to 1. A high risk for a pediatric SRBD is defined as positive responses to more than a third of the items [15].

### 2.4. Definition 

Underweight and normal weight, overweight, and obesity were determined by body mass index (BMI), which was calculated by dividing the students’ weight in kilograms by the square of their height in meters. Obesity and overweight were assessed according to the criteria defined by the international cutoff points for BMI by age and sex [16]. Recent weight, height, waist circumference, neck circumference, abdominal circumference, and hip circumference measurements were recorded and reported by parents and caretakers. Height and weight measurements were recorded to the nearest 1 cm and 1 kg. Neck circumference was measured below the laryngeal prominence with the measuring tape applied perpendicular to the long axis of the neck [17]. Waist circumference was measured at the level of the umbilicus. Hip circumference was measured at the level of maximum extension of the buttocks [18]. Measurements were taken to the nearest 1 cm. Self-reported exercise was divided into never exercise, sometimes exercise and often exercise. What we defined the snoring is someone snores more than half the time while sleeping [19]. Preterm birth was defined as children born before 37 weeks gestation; full-term birth was defined as children born from 37 completed weeks to less than 42 completed weeks; and post-term was defined as children born 42 completed weeks or more [20].

### 2.5. Statistical Analysis 

Data entry and statistical analysis were performed using Epidata (Version 3.1, Odense, Denmark) and SPSS (Version 21.0, IBM SPSS, IBM Corp, Armonk, NY, USA). Categorical variables were presented as frequency counts or percentages, and continuous variables were reported as medians and interquartile ranges. Chi-square test was used to describe the distribution of demographic characteristics, and Wilcoxon signed-rank test was used to compare the differences in continuous variables. Univariate and multivariate logistic regression was used to investigate the associations between SRBD, snoring and overweight as well as obesity. Associated factors significant at *p* < 0.10 in the univariate analysis were entered into forward stepwise multivariate logistic regressions adjusting for gender and age. *p* values < 0.05 were considered statistically significant. All tests were two-sided.

## 3. Results

In total, 1955 subjects were selected, of which 1825 participants met the inclusion and exclusion criteria and were finally analyzed in this study. The survey included 837 boys and 988 girls. The median age of the students was 14.50 years, ranging from 11 to 18 years. Of all these subjects, 783 were from rural regions and 1042 were from urban regions. Most of the subjects were Han Chinese, with only a few of minority ethnicity. A total of 68 out of 1825 students were considered to have a SRBD, and the prevalence of SRBD in our study was thus 3.7%. The prevalences of overweight and obesity were 12.6% and 4.9%, respectively. Based on the questionnaire answers, 3.3% of subjects snored. 

The prevalences of SRBD and snoring among adolescents are shown in Table 1. Subjects with SRBD were older than those without SRBD (*p* < 0.05). Urban subjects had a higher prevalence rate than rural ones (*p* < 0.05). A higher prevalence of SRBD was found in subjects who never exercised than those who exercised sometimes and often (*p* < 0.05). The snoring rate was slightly higher in males than in females (*p* < 0.05). The difference in snoring distribution by BMI classification was significant (*p* < 0.05). Additionally, snoring was less prevalent in subjects whose parents had a higher level of education (*p* < 0.05).

The differences in anthropometric parameters in subjects with and without SRBD and snoring are shown in Table 2. Higher weight and BMI values were found in the SRBD group than those in the non-SRBD group (*p* < 0.05). Height, neck circumference, waist circumference, abdominal circumference and hip circumference did not significantly differ between the SRBD group and non-SRBD group (*p* > 0.05). For snoring, weight and waist circumference were higher in subjects who snored than those in the non-snoring group (*p* < 0.05).

The results of the univariate analysis are presented in Table 3. SRBD and snoring served as the outcome variables, and demographic characteristics and anthropometric parameters severed as the independent variables. The regression results indicated that age and weight were correlated with risk of SRBD. Residence, educational level, exercise and birth history were also significantly associated with SRBD (*p* < 0.05). The results of univariate logistic regression showed that a larger waist circumference, highest parental educational level and BMI classification were significantly associated with snoring (*p* < 0.05). Gender, exercise, birth history were significant at *p* less than 0.10 for snoring.

Multivariate logistic regressions were used to analyze the factors influencing SRBD and snoring in Table 4. Residence, educational level, exercise, birth history and BMI classification significantly related to SRBD were entered into forward stepwise multivariable logistic regression, gender and age were adjusted. Urban residence (OR = 2.356, 95%CI: 1.251–4.435) was associated with an increased risk of SRBD compared with those living in rural areas. Post-term birth was significantly associated with SRBD, with an OR of 3.275 (1.396–7.683). We did not find a correlation between overweight or obesity and SRBD (OR = 0.969, 95%CI: 0.445–2.112; OR = 1.747, 95%CI: 0.655–4.658, respectively). In order to analyze the associated factors of snoring, we entered exercise, birth history, highest parental degree and BMI classification into forward stepwise multivariate logistic regression adjusting for gender and age. Association between overweight (OR = 2.063; 95%CI: 1.062–4.006) and snoring was found in this study. The regression results also showed that preterm birth had a correlation with snoring (OR = 2.255, 95%CI: 1.021–4.980). Parental education of a university degree and above was shown to be an influencing factor for snoring (OR = 0.265, 95%CI: 0.083–0.850).

## 4. Discussion

To describe the epidemiology of SRBD in northeast China and explore the relationships between SRBD, snoring and obesity and other factors in adolescents, we performed this survey of middle school students aged 11–18 years from urban and rural areas. To our knowledge, this was the first survey on SRBD focused on adolescents in Changchun City, Jilin Province. Although numerous reports on sleep have been published, only a few studies have reported on SRBD and snoring in adolescents in Mainland China, especially in Northeast China.

Based on our data, we identified that approximately 3.7% of adolescents in Changchun, Northeast China, were affected by SRBD. This result is consistent with the evaluated prevalence of 0%–5.7% in other studies on general children [21,22]. The snoring rate of 3.3% in this study agrees with the rates published in epidemiologic studies of 2.4% to 15.6% [14]. Nevertheless, a previous snoring rate of 20% has been reported in obese children by caretakers, and this prevalence is much higher than that in our population [23]. The difference in age ranges and populations between the studies were the principal reasons for the differential prevalences.

In this study, the age of SRBD group was higher than that of on-SRBD group, and SRBD were more common in students in senior middle school than those in junior middle school. The higher prevalence of SRBD in older students may occur partly because of the reduction in upper airway neuromuscular response with age, even among adolescents in different Tanner stages [24]. According to the results, a higher prevalence of SRBD was observed in never exercise group. Davis et al. found that regular exercise could improve snoring in overweight children and reduced the risk of SRBD [25]. A study has highlighted that children with a higher than normal weight were more likely to experience SRBD [5]. We also found that a higher weight in the SRBD group compared to those in the non-SRBD group. In weighty populations, breathing is not smooth during sleep, which may result in sleep apnea [19]. Sánchez-Armengol et al. indicated that healthcare interventions might have a beneficial effect on snoring by avoiding weight gain in adolescents [26].

The results of multivariate analysis demonstrated that urban residence and post-term birth were significantly correlated with SRBD, and preterm birth and highest parental degree (university and above) as well as overweight played a critical role in snoring. Liu et al. found that urban residence presented as a risk factor, which is similar to our results [27]. The main reasons for this association might be the underlying environmental and lifestyle differences between urban and rural areas. Several published surveys that examined whether birth history influenced SRBD demonstrated conflicting results. Goldstein et al. revealed that preterm birth had an important role in snoring [14]. Their results were similar to ours. This influence might be due to upper airway size and development of respiratory control [28]. Post-term birth was found to be a risk factor for SRBD compared to full-term birth. The main reason for this finding might be the different classification criterion used for birth history, which we divided into three segments; further studies are needed to explore the reasons underlying this association. This survey also found that a highest parental degree of university and above was related to snoring. This finding conflicted with the results of a survey in which parental educational level had no potential effect on snoring in children [29]. However, Friberg et al. found that a low level of maternal education was a risk factor for SRBD in offspring [30]. 

Previous surveys that estimated the associations of overweight and obesity with SRBD reported a positive correlation. Although the relationship between obesity and SRBD has been described in adults, controversy remains regarding the association in children [29,31,32]. A study on children by Redline et al. revealed that obesity was a risk factor for SRBD, with an odds ratio of 4.59 [33]. Wing et al. reported that SRBD occurred in 26%–33% of obese children [29]. The decrease in lung volume in obese children was previously shown to lead to an increasing prevalence of SRBD [34]. Su et al. found that school children with obesity had higher obstructive apnea-hypopnea index (OAHI) than those without obesity [35]. Nevertheless, the opposite finding was presented in a study by Sardón et al. [31]. After conducting the multivariate logistic regression analysis, we failed to observe a relationship between obesity and SRBD in the current study. This finding was consistent with a study by Goldstein et al. [14]. One of reason for the lack of association might be the lower rate of obesity. In this study, the prevalence of obesity (4.7%) was lower than the 5.3% identified among children 7–18 years old in the China Health and Nutrition Survey [36]. Although obese children are prone to suffering from SRBD, the limited number of obese adolescents in the current study may have affected the outcome. Second, intact upper airway reflexes can protect obese adolescents from SRBD [37]. Third, different ethnicities and districts might account for this finding [12,38,39,40]. Kohler et al. demonstrated that obesity was a significant predictor of upper airway obstruction in Australian Caucasian children [38]. Lam et al. reported obesity was related to severity of OSA in children in Hong Kong, although the correlation is mild [40]. Finally, in our adolescent group, adenotonsillar hypertrophy may have played a role in the occurrence of SRBD, which would have affected our results.

Adenotonsillar hypertrophy has been identified as the most common cause of OSA in children [41]; however, with the decline in lymphoid tissue growth with age, obesity becomes an increasingly important factor for sleep apnea and snoring from childhood to adolescence [11], and overweight is found in a majority of OSA patients [42]. A relation between overweight and snoring was showed in the present study. Fat deposits in upper airway tissues might narrowed upper airway [43], which causes air turbulence and vibrations and might generate snoring during sleep [44]. Therefore, weight control is essential to prevent the development of SRBD in adolescents. 

China has undergone tremendous changes since the economic reform and opening-up policy. The rapid development of the economy and urbanization process transformed our lifestyles, affecting aspects from food and clothes to universal health [45]. However, these lifestyle transitions and the interactions between environmental and genetic factors have resulted in different health problems such as obesity, diabetes, and sleep problems et al. Childhood SRBD may be a risk factor for later development of adult sleep apnea [46]. Adolescents is crucial in the development of SRBD because hormone changes during this period. Sánchez-Armengol and colleagues conducted a 4-year follow-up study and demonstrated that habitual snoring tended to persist during adolescents if no intervention was undertaken [26]. In the present study, we found overweight, residence, birth history and parental education level were correlated with snoring and SRBD in adolescents. The findings of this study may have implications for the long-term management of SRBD and the intervention that has to be undertaken in order to modify its natural history. In northeast China, the living habits and diet structure need to be changed for losing weight. The Chinese government should pay more attention to researches of the relative diseases, enhance public awareness of healthy lifestyles, strengthen the infrastructure, and promote basic public health services to lay a foundation for disease prevention and control.

There are several limitations to this study. First, we used the PSQ to assess SRBD instead of polysomnography (PSG), which is the gold standard for diagnosing SRBD. It is inconvenient for such a large number of adolescents to enter a sleep lab and complete an overnight PSG for the overburden of homework every day in China. Second, genetic factors and socioeconomic factors were not included in our questionnaire, and we therefore did not assess their influence on SRBD and snoring. However, this study also had several strengths. This was the first investigation to focus on SRBD and snoring in students 11–18 years in Changchun, Jilin Province. Furthermore, we estimated the epidemiological factors affecting SRBD and snoring in adolescents, thus providing a meaningful basis to design control measures to decrease the incidence of these conditions.

## 5. Conclusions 

In this cross-sectional study, the prevalences of SRBD and snoring were similar to those previously reported. Urban areas and post-term birth were important influencing factors for SRBD; overweight, highest parental degree (university and above) and preterm birth were key factors for snoring in adolescents. SRBD and snoring is a concern in adolescents with risk factors of urban residence, pre-term birth, low parental education, or an overweight BMI. Further research should be conducted.

## Figures and Tables

**Table 1:** Prevalence of SRBD and snoring among adolescents (*n* = 1825).

Variables	SRBD	Non-SRBD	χ^2^/Z	*p*	Snoring	Non-Snoring	χ^2^/Z	*p*
	*n* (%)	*n* (%)			*n* (%)	*n* (%)		
Gender			1.425	0.233			3.886	0.049
Male	36 (4.3)	801 (95.7)			35 (4.2)	802 (95.8)		
Female	32 (3.2)	956 (96.8)			25 (2.5)	963 (97.5)		
Age (years)	15.50 (13.63–16.51)	14.50 (13.50–15.50)	−2.397	0.017	15.00 (13.75–16.26)	15.30 (13.91–16.07)	−0.156	0.876
Residence			6.455	0.011			0.111	0.739
Rural	19 (2.4)	764 (97.6)			27 (3.4)	756 (96.6)		
Urban	49 (4.7)	993 (95.3)			33 (3.2)	1009 (96.8)		
Ethnicity			0.458	0.499			0.332	0.564
Han	68 (3.8)	1724 (96.2)			60 (3.3)	1732 (96.7)		
Others	0 (0.0)	33 (100)			0 (0)	33 (100)		
Educational level			7.519	0.006			0.081	0.776
Junior middle school	23 (2.5)	892 (97.5)			29 (3.2)	886 (96.8)		
Senior middle school	45 (4.9)	865 (95.1)			31 (3.4)	879 (96.6)		
Exercise			7.552	0.023			5.634	0.060
Never	26 (5.8)	425 (94.2)			21 (4.7)	430 (95.3)		
Sometimes	12 (2.5)	466 (97.5)			9 (1.9)	469 (98.1)		
Often	30 (3.3)	866 (96.7)			30 (3.3)	866 (96.7)		
Highest parental degree			0.975	0.807			11.637	0.009
Primary school or low	5 (5.3)	90 (94.7)			5 (5.3)	90 (94.7)		
Junior high school	28 (3.5)	771 (96.5)			22 (2.8)	777 (97.2)		
Senior high school	19 (4.1)	449 (95.9)			25 (5.3)	443 (94.7)		
University and above	16 (3.5)	447 (96.5)			8 (1.7)	455 (98.3)		
Birth history			6.278	0.043			5.689	0.058
Full-term birth	46 (3.3)	1365 (96.7)			39 (2.8)	1372 (97.2)		
Preterm birth	6 (4.5)	127 (95.5)			8 (6.0)	125 (94.0)		
Post-term birth	7 (9.9)	64 (90.1)			4 (5.6)	67 (94.4)		
BMI classification			0.926	0.630			6.564	0.038
Under and normal weight	54 (3.6)	1446 (96.4)			44 (2.9)	1456 (97.1)		
Overweight	9 (3.9)	221 (96.1)			14 (6.1)	216 (93.9)		
Obesity	5 (5.6)	85 (94.4)			2 (2.2)	88 (97.8)		

**Table 2:** Anthropometric parameters of adolescents.

Variables	SRBD	Non-SRBD	Z	*p*	Snoring	Non-Snoring	Z	*p*
	M (Q1–Q3)	M (Q1–Q3)			M (Q1–Q3)	M (Q1–Q3)		
Height (cm)	165.00 (162.00–173.00)	165.00 (160.00–172.00)	−1.268	0.205	165.00 (160.00–175.00)	165.00 (160.00–172.00)	−0.640	0.522
Weight (kg)	58.00 (50.25–67.00)	53.00 (47.00–61.00)	−3.232	0.001	55.50 (50.00–69.75)	53.00 (47.00–61.00)	−2.042	0.041
Neck circumference (cm)	32.00 (30.25–34.75)	32.00 (31.00–34.00)	−0.611	0.541	32.00 (32.00–36.00)	32.00 (31.00–34.00)	−1.805	0.071
Waist circumference (cm)	75.50 (72.00–83.75)	74.00 (70.00–80.00)	−1.193	0.233	78.00 (72.00–84.50)	74.00 (70.00–80.00)	−2.033	0.042
Abdominal circumference (cm)	78.00 (75.00–85.75)	78.00 (74.00–85.00)	−0.519	0.604	78.00 (71.25–90.00)	78.00 (74.00–84.50)	−0.474	0.635
Hip circumference (cm)	90.00 (85.00–95.00)	90.00 (86.00–95.00)	−0.346	0.730	90.00 (88.25–95.00)	90.00 (86.00–95.00)	−1.104	0.269
BMI (kg/m^2^)	20.68 (18.44–23.44)	19.47 (17.58–21.97)	−2.889	0.004	20.22 (18.19–24.31)	19.49 (17.58–21.97)	−1.803	0.071

**Table 3:** Univariate logistic regression analyses on the influencing factors for SRBD and snoring.

Variables	SRBD		*p*	Snoring		*p*
	OR	95%CI		OR	95%CI	
Gender						
Male	1			1		
Female	0.745	0.458–1.210	0.234	0.595	0.353–1.002	0.051
Age (years)	1.225	1.039–1.444	0.016	0.966	0.812–1.149	0.695
Residence						
Rural	1			1		
Urban	1.984	1.159–3.398	0.013	0.916	0.546–1.536	0.739
Educational level						
Junior middle school	1			1		
Senior middle school	2.018	1.210–3.363	0.007	1.077	0.644–1.803	0.776
Exercise						
Never	1			1		
Sometimes	0.421	0.210–0.845	0.015	0.393	0.178–0.867	0.021
Often	0.566	0.331–0.970	0.038	0.709	0.401–1.254	0.237
Highest parental degree						
Primary school or low	1			1		
Junior high school	0.654	0.246–1.735	0.393	0.510	0.188–1.379	0.184
Senior high school	0.762	0.277–2.093	0.598	1.016	0.379–2.724	0.975
University and above	0.644	0.230–1.804	0.403	0.316	0.101–0.990	0.048
Birth history						
Full-term birth	1			1		
Preterm birth	1.402	0.587–3.346	0.447	2.251	1.030–4.924	0.042
Post-term birth	3.246	1.410–7.471	0.006	2.100	0.729–6.049	0.169
BMI classification						
Under and normal weight	1			1		
Overweight	1.090	0.531–2.240	0.813	2.145	1.156–3.980	0.016
Obesity	1.575	0.614–4.040	0.344	0.752	0.179–3.153	0.697
Height	1.019	0.992–1.048	0.172	1.015	0.985–1.045	0.336
Weight	1.024	1.008–1.041	0.003	1.018	1.000–1.036	0.053
Neck circumference (cm)	1.043	0.960–1.133	0.323	1.087	0.999–1.182	0.053
Waist circumference (cm)	1.016	0.992–1.039	0.187	1.027	1.004–1.052	0.023
Abdominal circumference (cm)	1.014	0.991–1.038	0.240	1.011	0.986–1.036	0.403
Hip circumference (cm)	0.995	0.971–1.020	0.682	1.019	0.991–1.048	0.176

**Table 4:** Multivariate logistic regression analyses of risk for SRBD and snoring.

Variables	SRBD		*p*	Snoring		*p*
	OR	95%CI		OR	95%CI	
BMI classification						
Under and normal weight	1			1		
Overweight	0.969	0.445–2.112	0.937	2.063	1.062–4.006	0.032
Obesity	1.747	0.655–4.658	0.265	0.357	0.048–2.674	0.316
Residence						
Rural	1			-	-	
Urban	2.356	1.251–4.435	0.008	-	-	
Birth history						
Full-term birth	1			1		
Preterm birth	1.414	0.586–3.410	0.441	2.255	1.021–4.980	0.044
Post-term birth	3.275	1.396–7.683	0.006	2.540	0.862–7.484	0.091
Highest parental degree						
Primary school or low	-	-		1		
Junior high school	-	-		0.390	0.139–1.092	0.073
Senior high school	-	-		0.738	0.264–2.061	0.561
University and above				0.265	0.083–0.850	0.026

Note: adjusted for age and gender.
